# Comparative study of mental health and quality of life in long term refugees and host populations in Oru-Ijebu, Southwest Nigeria

**DOI:** 10.1186/1756-0500-5-394

**Published:** 2012-07-31

**Authors:** Oluwaseun O Akinyemi, Eme T Owoaje, Olusimbo K Ige, Oluwafemi A Popoola

**Affiliations:** 1 Department of Community Medicine, University College Hospital, Ibadan, Nigeria; 2 Department of Community Medicine, College of Medicine, University of Ibadan, Ibadan, Nigeria

**Keywords:** Mental health, Quality of Life, Refugees, Nigeria

## Abstract

**Background:**

Refugees as “People Living in Highly Stressful Situation” are particularly vulnerable to mental ill-health as a result of the trauma experienced pre- and post-migration. The lack of information on the mental health disparities of refugees and non-refugees in West Africa is what this study aimed to bridge.

A cross-sectional study design was employed using a cluster sampling technique. Interviewer-administered structured questionnaires consisting of the Mini-International Neuropsychiatric Interview (MINI), WHO quality of life (WHOQOL-BREF) and the Community Quality of Life (CQoL) were used for data collection. Data were analyzed with SPSS version 17. Logistic regression analysis was used to determine the predictors of mental health status and QoL.

**Results:**

Respondents consisted of 444(45.7%) refugees and 527(54.3%) non-refugees. Two-thirds 292 (66%) of the refugees were Liberians. Mean age: refugees - 34.8 ± 12.8 years versus non-refugees - 33.3 ± 8.1 years (p < 0.05). While the majority 376(84.7%) of the refugees were married, most 468(88.8%) of the native population were not (p < 0.001). Significantly higher proportion of refugees had polygamous marriages, lived in poorer type of accommodation and had no formal education compared to the non-refugees (p < 0.05). The overall QoL and CQoL scores were both significantly lower for the refugees (p < 0.001). Refugees were three times more likely than non-refugees to have poor mental health [OR: 3.43; 95%CI: 1.83-6.40]. Overall, being currently ill tripled the odds of mental ill health [OR: 2.73; 95%CI: 1.98-3.77]. Unskilled workers [OR: 2.78; 95%CI: 1.68-4.60], skilled workers [OR: 2.98; 95%CI: 2.03-4.38] and the unemployed [OR: 1.94; 95%CI: 1.29-2.92] had two or more times the odds of poor mental health compared to professionals.

**Conclusions:**

QoL and occupational status were the major threats to the mental health of the refugees. Results of this study point to the need for continued attention to not only the healthcare needs but the welfare, housing, employment and overall QoL to support the long-term mental health of refugees and non-refugee populations alike.

## Background

Eliminating health disparities and increasing the quality and years of healthy life for all people is a global goal. To achieve these, identifying and addressing the health needs of populations that are often overlooked need to be prioritised. Refugees as “People Living in Highly Stressful Situation” are particularly vulnerable to mental ill-health as a result of the trauma experienced pre- and post-migration [1-3]. Although literature abounds on the mental health disparities of refugees and non-refugees in many regions of the world, the lack of information on refugee communities in West Africa is notable [4]. In a meta-analysis of pre- and post- migration factors associated with mental ill-health among refugees, only two of the 59 independent comparisons of refugee and non-refugee populations in adults from the same low-income country were compared and none was from Africa [5]. Although Africans constitute only 12 per cent of the global population [6], about a quarter of the world’s 8.8 million refugees are in Africa; 150,000 of whom live in West Africa [6].

Apart from the trauma which drove many into exile post migration factors have also been linked to the excess burden of mental health problems borne by refugees [7]. Therefore, increasing attention is now being drawn to the conditions in which refugees live in exile and the long-term mental health of refugees in exile. The mental health status of refugees in Sub-Saharan Africa is of particular interest because of the poorer quality of life (QoL) of the region compared to other regions of the world [8]. The QoL, though subjective as an indicator of well-being has been shown to be inextricably associated with the mental health status [1,3]. It is yet to be documented if refugees in host communities with similar culture and living conditions fare better than those in richer countries. The added dimension of the community quality of life (CQoL) which is the perception of “being”, “belonging” and “becoming” a part of the community in which one lives may also have consequences for mental morbidity [3]. The research questions in focus in the present study are: Do disparities exist in the mental health status of refugees and non-refugee populations in Nigeria? Would the QoL and CQoL also differ and would such disparities be sufficient to threaten the mental well-being of refugees in excess of the non-refugee population? This study aimed at providing community-based comparative assessment data on the mental health status and its interaction with the QoL and CQoL of refugees who have been long term residents in Oru-Ijebu South West Nigeria compared with the non-refugee population. The study’s hypothesis was: there is no difference in the mental health and QoL of refugees and non-refugees in Southwest Nigeria.

## Methods

### Study area

This study was conducted in Oru-Ijebu, Ogun State, South-western Nigeria. Oru-Ijebu, which borders Ago-Iwoye, a university town, is a semi-urban town which according to the 2006 census had a population of 27,000 [9]. The Oru Refugee Camp is located on the outskirts, about 500 m to Oru town. The Camp which is the only one in the country was established in October 1990 as an initiative of the United Nations High Commissioner for Refugees (UNHCR) with the approval of the Federal Government of Nigeria and in collaboration with the Nigerian Red Cross Society [9,10]. The camp was established as an aftermath of the Liberian civil war in 1989 and many of the refugees have been resident in the camp since then. Nationalities represented in the Camp included Liberians, Sierra-Leoneans and Togolese. Health and social welfare benefits were withdrawn and the camp was officially closed in 2007 in order to encourage voluntary repatriation of the refugees. Many of the refugees however chose to remain in the camp. There are about 5,000 refugees currently living in the camp, majority of whom are Liberians. The Camp is made up of 11 residential blocks consisting of 72 houses of two bedroom units. Many other makeshift mud houses have been constructed by the refugees over the years. There is a non-functional mini clinic and one primary school of eight classrooms. Some of the refugees were involved in vocations like hairdressing, trading, transportation using motorcycles, teaching and clerical work within and outside the camp.

### Study population

The study population comprised of male and female residents of Oru community and the refugee camp aged 18 years and above who have resided in the area for at least one year prior to the study.

Ethical approval for this study was obtained from the University of Ibadan/University College Hospital Institutional Review Committee before study was commenced. Written informed consent was also obtained from participants before administering questionnaires. A cross-sectional study design was used comparing refugees with non-refugees within the same geographical location. The minimum sample size estimated to compare the proportions with poor mental health status was based on a prior estimate of 22% for poor mental health [11]. An expected difference of 15% was used, at 95% confidence interval, 80% power and 15% precision. This was adjusted by a factor of two for clustering effect; and allowance was made for 10% non-response. A sample size of 431 per group was thus estimated. A cluster sampling technique was used to obtain a representative sample of the refugee and non-refugee communities. Adults aged 18 years and above were selected from the Oru community and the refugee camp. For the non-refugees, four enumeration areas (clusters) were chosen by balloting from the 60 enumeration areas in Oru-Ijebu. Each enumeration area had 15-30 houses with about 5-11 adults per house. All eligible adults in each enumeration area were interviewed. For the refugees, eight residential blocks were selected by balloting from the 11 blocks in the refugee camp. All eligible respondents in the 10 houses (each consisting of four to five two-bedroom units) present in each selected block were interviewed.

### Study instruments

Interviewer-administered structured questionnaires consisting of the Mini-International Neuropsychiatric Interview (MINI) [12], WHO quality of life (WHOQOL-BREF) [13] and the Community Quality of Life adapted from the Florida MAPP Field Guide [14], were used for data collection. The Cronbach’s alphas were 0.86, 0.93 and 0.93 respectively. The assessment of clinical variables such as suicide ideation, visual hallucination, drug and alcohol abuse, mania, posttraumatic stress disorder (PTSD), obsession and depression was done using respondents’ responses to specific questions on the MINI.

### Measures

#### Quality of life

The World Health Organization quality-of-life group defined health-related quality of life as the individual’s subjective evaluation of disease and impairment and disabilities [13]. The quality of life was measured with the WHOQOL-BREF with 26 questions which were measured on a 5 point Likert scale. Scores were scaled in a positive direction (i.e. higher scores denoting higher quality of life) and summed. The WHOQOL-BREF was developed to be applicable in diverse cultural settings and was designed across several countries including one in Sub-Saharan Africa [15].

#### Mental health

Defined as a state of well-being in which every individual realizes his or her own potential, can cope with the normal stresses of life, can work productively and fruitfully, and is able to make a contribution to her or his community [16]. Mental health was assessed using the Mini-International Neuropsychiatric Interview (MINI). MINI has been used in a similar environment and validated in Nigeria, the interrater reliability (Cohen’s kappa) was 0.86 [17]. The MINI consists of 26 questions with “Yes” or “No” responses. “Yes” was scored one and “No” zero (0). Scores were summed and Score ≤ 5 were labeled as green zone (good mental health), scores 6-8 fell into the yellow zone (borderline) and scores 9-24 fell in the red zone (poor mental health). These three zones were dichotomised for the purpose of logistic regression analysis; the Green zone, and the Red (i.e. a combination of the Yellow and Red zones [13,18].

#### Community quality of life

Is the assessment of the quality of life within a community – understanding a community from the members’ point of view [19]. Community QoL was assessed using the Community QoL questionnaire. The instrument consisted of 12 questions which elicits responses through a Likert scale (i.e., 1 to 5 with 1 being low and 5 being high) [14] Each participant’s score was summed.

#### Disability

Defined as any impairment that can make performing an everyday task more difficult. Disability and physical illness were assessed through self-report.

### Data analysis

Data was collected in August 2010 and analyzed with SPSS version 17. The mean scores for the QoL and CQoL were compared with the Student *t*-test. The determinants of mental health status and QoL were explored using logistic regression analyses. The -2Log Likelihood value was used to assess what model had the best fit. Results are presented as odds ratios (OR) with 95% confidence limits.

## Results

### Socio-demographic characteristics of respondents

Response rate was 98.9%, consisting of 444(45.7%) refugees and 527(54.3%) non-refugees. The mean length of stay of refugees in the Camp was 8.6 ± 4.8 years. Table 1 shows the socio-demographic characteristics of respondents. The refugees were slightly older with a mean age of 34.8 ± 12.8 years versus 33.3 ± 8.1 years for the non-refugee population (p < 0.05). The sex distribution was not significantly different. While the majority 376(84.7%) of the refugees were married, most 468(88.8%) of the native population were not (p < 0.001). Significantly higher proportion of refugees had polygamous marriages, lived in poorer type of accommodation and had no formal education compared to the non-refugees. The occupations of both populations did not differ significantly.

**Table 1:** Socio demographic characteristics of respondents

**Variable**	**Refugee (N = 444)**	**Non-refugee (N = 527)**	***χ***^**2**^	**p-value**
	**Frequency (%)**	**Frequency (%)**		
**Age (years)**				
< 25	97 (21.8)	61 (11.6)		
25-34	167 (37.6)	286 (54.3)	55.7	<0.001
35-44	90 (20.3)	134 (25.4)		
**> =** 45	90 (20.3)	46 (8.7)		
**Sex**				
Male	263 (59.2)	320 (60.7)	0.22	0.64
Female	181 (40.8)	207 (39.3)		
**Marital status**				
Never Married	13(2.9)	468(88.8)		
Married	376(84.7)	48(9.1)	711.6	<0.001
Divorced	28(6.3)	5(0.9)		
Widowed	27(6.1)	6(1.1)		
**Family type**	**n = 376**	**n = 48**		
Monogamous	256 (68.1)	37 (77.1)	7.9	0.007
Polygamous	120 (31.9)	11 (22.9)		
**Religion**				
Christianity	310 (69.8)	383 (72.7)		
Islam	134 (30.2)	144 (27.3)	0.962	0.345
**Actively Religious**				
Yes	314(70.7)	467(88.6)	49.03	<0.001
No	130(29.3)	60(11.4)		
**Housing**				
One room	399(89.9)	254(48.2)	189.96	<0.001
Two or more rooms	45 (10.1)	273(51.8)		
**Educational status**				
No formal education	64(14.4)	13(2.5)	138.56	<0.001
Primary education	42(9.5)	18(3.4)		
Secondary education	232(52.3)	190(36.1)		
Tertiary education	106(23.9)	306(58.1)		
**Occupation**				
Professional	133(30.0)	141(26.8)	4.94	0.294
Skilled worker	44(9.9)	68(12.9)		
Student	92(20.7)	122(23.1)		
Unemployed	48(10.8)	44(8.3)		
Unskilled worker	127(28.6)	152(28.8)		

### Ethnicity of indigenes and the nationality of refugees

Two-thirds, 292 (66%), of the refugees were Liberians, other nationalities represented were Sierra-Leoneans, 143 (33%), and Togolese, 3 (1%). Of the non-refugees, majority 427 (81%) were of Yoruba ethnicity.

### Health status by respondent status

Table 2 shows health status by respondent category. A significantly higher proportion of non-refugee respondents reported physical disability like chronic back pain and joint pain, 159(30.2%) versus 84(18.9%) (p < 0.001). There was however no statistically significant difference between refugees, 151(43.0%), and non-refugees, 209(39.7%), who reported current health problems (p > 0.05).

**Table 2:** Health status by respondent category

**Variable**	**Refugee (N = 444)**	**Non-refugee (N = 527)**	***χ***^**2**^	**p-value**
	**Frequency (%)**	**Frequency (%)**		
**Physical disability**				
Yes	84(18.9)	159(30.2)	16.3	p < 0.001
No	360(81.1)	368(69.8)		
**Current illness**				
Yes	151(34.0)	209(39.7)	3.3	0.069
No	293(66.0)	318(60.3)		

### Disparities in the quality of life (QoL) and CQoL profile of respondents

The QoL profile of respondents based on scores from the WHOQOL-BREF is shown in Table 3. The mean QoL scores for each of the four domains examined were significantly lower for the refugees. The overall QoL and CQoL scores were both significantly lower for the refugees (p < 0.001).

**Table 3:** Quality of Life (QoL) profile of respondents

**Variable**	**N**	**Mean ± SD**	***T*** **test**	**p-value**
**QoL domains**				
**Physical health**				
Refugee	444	19.45 ± 4.18	17.14	<0.001
Non-refugee	527	23.83 ± 3.78		
**Psychological**				
Refugee	444	16.86 ± 4.04	17.16	<0.001
Non-refugee	527	20.98 ± 3.44		
**Social relationship**				
Refugee	444	8.66 ± 2.59	14.86	<0.001
Non-refugee	527	10.87 ± 2.04		
**Environment**				
Refugee	444	26.09 ± 5.03	29.08	<0.001
Non-refugee	527	26.09 ± 4.57		
**Total QoL Score**				
Refugee	444	67.47 ± 13.46	25.89	<0.001
Non-refugee	527	89.47 ± 2.95		
**CQoL Score**				
Refugee	444	21.64 ± 5.54	15.15	<0.001
Non-refugee	527	30.64 ± 11.44		

### Mental health disparities

**Figure 1:**
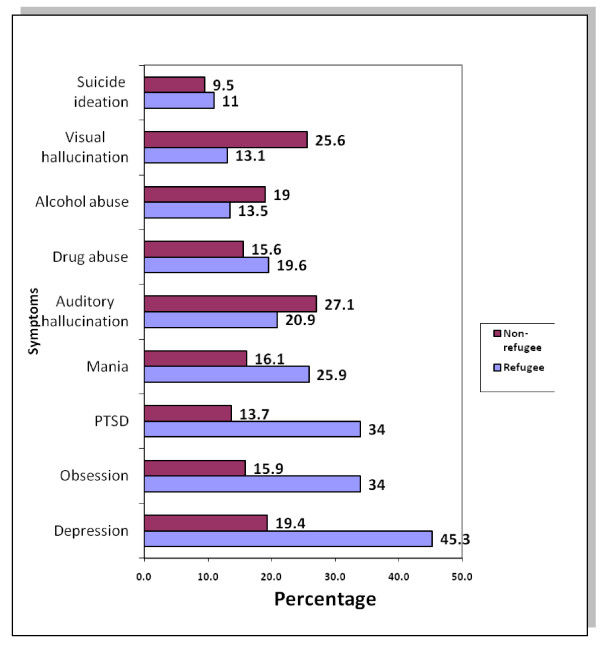
Proportion of respondents reporting mental health disorders by status.

Figure 1 shows the reported symptoms of mental ill-health as assessed by the MINI. The most commonly reported symptoms among the refugees was depression (45.3%), p < 0.05, followed by obsession (34%), p < 0.05, Post Traumatic Stress Disorder (34%), p < 0.05 and mania (25.9%). Suicidal ideation was the least reported (11%), p > 0.05, whereas auditory hallucination (27.1%), visual hallucination (25.6%) and alcohol abuse (19%) were commoner among the refugee population (p < 0.05).

### Mental health profile of respondents

Table 4 shows the mental health profile of respondents. A significantly higher proportion of the refugees, 275(61.9%), were in the red zone compared to the non-refugees 183 (34.7) (p < 0.001).

**Table 4:** Mental health profile of respondents

**Variable**	**Refugee (N = 444)**	**Non-refugee (N = 527)**	***χ***^**2**^	**p-value**
	**Frequency (%)**	**Frequency (%)**		
**MINI Zone**				
Green	169 (38.1)	344 (65.3)	71.6	<0.001
Yellow	126(28.4)	85(16.1)		
Red	149 (33.6)	98 (18.6)		

### Predictors of poor mental health status among respondents

The predictors of poor mental health following logistic regression analysis are presented in Table 5. Refugees were three times more likely than non-refugees to have poor mental health. Being currently ill tripled the odds of mental ill health. Unskilled workers, skilled workers and the unemployed had two or more times the odds of poor mental health compared to professionals.

**Table 5:** Factors associated with poor mental health among all respondents

**Characteristics**	**Odds ratio**	**95% Confidence**	**p-value**
		**Interval**	
**Age (years)**			
< 25	0.54	0.29 – 1.01	0.055
25-34	0.86	0.52 – 1.41	0.548
35-44	1.14	0.69 – 1.90	0.611
> = 45	1		
**Sex**			
Male	1.07	0.80 – 1.43	0.622
Female	1		
**Family type**			
Monogamous	0.76	0.56 – 1.04	0.083
Polygamous	1		
**Religion**			
Christianity	0.90	0.65 – 1.24	0.516
Islam	1		
**Actively Religious**			
Yes	1.08	0.74 – 1.58	0.692
No	1		
**Housing**			
One room	0.72	0.49 – 1.05	0.087
Two or more rooms	1		
**Educational status**			
No formal education	0.81	0.43 – 1.54	0.517
Primary education	0.83	0.41 – 1.64	0.584
Secondary education	1.39	0.95 – 2.04	0.088
Tertiary education	1		
**Marital status**			
Married	1.86	1.29 – 2.68	0.001
Never married	1.73	1.08 - 2.77	0.018
Divorced/widowed	1		
**Occupation**			
Unskilled worker	2.78	1.68- 4.60	<0.001
Unemployed	1.94	1.29- 2.92	0.001
Student	0.33	0.77-2.21	0.33
Skilled worker	2.98	2.03- 4.38	<0.001
Professional	1		
**Status**			
Refugees	3.43	1.83 – 6.40	<0.001
Non-refugees	1		
**Current illness**			
Yes	2.73	1.98 – 3.77	<0.001
No	1		
**QoL Score**	0.98	0.97- 0.99	0.001
**CQoL Score**	1.03	1.01-1.05	0.001

The determinants of poor mental health among refugees are shown in Table 6. Being engaged in unskilled occupations significantly increased the likelihood of poor mental health. The likelihood of poor mental health decreased by 0.9 for each rise in QoL score.

**Table 6:** Factors associated with poor mental health among refugees

**Characteristics**	**Odds ratio**	**95% Confidence**	**p-value**
		**Interval**	
**Age (years)**			
< 25	0.87	0.37 – 2.01	0.737
25-34	1.59	0.81 – 3.14	0.180
35-44	1.79	0.90– 3.57	0.098
> = 45	1		
**Sex**			
Male	0.87	0.57 – 1.32	0.501
Female	1		
**Family type**			
Monogamous	0.90	0.59 – 1.39	0.644
Polygamous	1		
**Religion**			
Christianity	0.91	0.59 – 1.40	0.658
Islam	1		
**Actively Religious**			
Yes	0.82	0.51– 1.32	0.417
No	1		
**Housing**			
One room	0.69	0.35– 1.38	0.295
Two or more rooms	1		
**Educational status**			
No formal education	0.78	0.37 – 1.66	0.522
Primary education	0.867	0.37 – 2.02	0.741
Secondary education	0.96	0.56 – 1.67	0.889
Tertiary education	1		
**Marital status**			
Married	1.16	0.63 – 2.14	0.630
Never married	1.34	0.73 - 2.47	0.348
Divorced/widowed	1		
**Occupation**			
Unskilled worker	2.19	1.04- 4.61	0.045
Unemployed	2.20	1.23- 3.93	0.008
Student	0.91	0.46- 1.81	0.796
Skilled worker	2.82	1.64- 4.85	<0.001
Professional	1		
**Current illness**			
Yes	1.77	1.12 – 2.78	0.014
No	1		
**QoL Score**	0.98	0.96-0.99	0.001
**CQoL Score**	0.95	0.56 -1.62	0.853

The factors associated with poor mental health among the non refugee population are shown in Table 7. Risk factors were: increasing age, male gender, current illness, being religious, poorer QoL scores, higher CQoL scores and employment as an unskilled or skilled worker.

**Table 7:** Factors associated with poor mental health among non-refugees

**Characteristics**	**Odds ratio**	**95% Confidence**	**p-value**
		**Interval**	
**Age (years)**			
< 25	0.28	0.09 – 0.87	0.029
25-34	0.25	0.10 – 0.64	0.004
35-44	0.37	0.14– 0.93	0.035
> = 45	1		
**Sex**			
Male	1.67	1.05 - 2.64	0.030
Female	1		
**Family type**			
Monogamous	0.60	0.37 – 0.97	0.035
Polygamous	1		
**Religious activities**			
Religious	3.55	1.47- 8.56	0.005
Not religious	1		
**Housing**			
One room	0.78	0.46– 1.32	0.350
Two or more rooms	1		
**Current illness**			
Yes	5.40	3.28 - 8.90	<0.001
No	1		
**QoL**	0.97	0.95-0.99	0.001
**CQoL**	1.03	1.01-1.05	0.013
**Marital status**			
Married	2.30	1.28 – 4.18	0.006
Never married	0.67	0.24 – 1.87	0.450
Divorced/widowed	1		
**Occupation**			
Unskilled Worker	3.49	1.64- 7.41	0.001
Unemployed	1.51	0.79 -2.90	0.214
Student	2.05	0.89- 4.71	0.93
Skilledworker	2.92	1.59- 5.38	0.001
Professional	1		

## Discussion

After an average stay of about a decade in exile, there persisted marked disparities between the mental health status of refugees and non-refugees in Nigeria. These findings correspond to those of previous studies of refugee and non-refugee populations [7,20,21]. The finding that the non-refugee population is very highly educated could be due to the proximity of the study area to a university town, thus a good number of the residents of Oru-Ijebu are university students and workers. The high prevalence of manic symptoms, obsessive compulsive symptoms, depression and Post Traumatic Stress Disorder (PTSD) among refugees in excess of indigenes has also been documented [6,16,22-24]. Although the prevalence of psychiatric symptoms (visual and auditory hallucinations) in this study was higher than previously reported in the country [25], they are however similar to reports from developed countries [26,27]. The reason for this observation is unclear though it may be due to differences in methodologies as MINI is a screening tool designed particularly for the use of non-specialised interviewers [12,28,29].

Disparities also emerged in the QoL and CQoL across all domains. This replicates findings among refugee populations in the USA and Sweden [30-32]. The observed disparity in mental health status persisted after adjusting for QoL, CQoL and socio-demographic variables. While better QoL emerged as protective as seen in other studies [13,30,33-36], increasing CQoL scores appeared to increase the probability for mental health problems. This is at variance from what has been previously documented [2,11,37]. The explanation for this is not clear but might be indicative of a sense of collective social suffering. However the marginal increased probability of mental ill health precludes firm conclusions and warrants further evaluation. Physical health status, marital status and employment status emerged as important covariates for all respondents. The impact of poor physical health and lower occupational status on both refugee and indigenous study groups is consistent with reports from other authors [34,38-40].

Within group evaluation revealed that the influence of QoL on both populations was comparable although the adverse impact of higher CQoL scores on mental health was restricted to the non-refugee population. Unlike the refugees many other factors such as increasing age, male gender and being religious affected the mental health of the native population. An overlap in the mental and physical functions was also evident among the native population. The risk factors for the indigenous population were similar to the findings of a previous Nigerian study [34].

Surprisingly, non-refugees reported more physical disability (although not statistically significant) compared with the refugees. A possible explanation for this observation could be the healthy worker’s bias [41-43]. Refugees could in fact be healthier than the native population, for them to have survived long distances from their countries of origin under adverse conditions whereas more disabled non-refugees are not able to move and leave their countries. This observation could also be due to sample selection bias [44,45].

Unlike other reports [5], acculturative stressors (CQoL) were not associated with greater mental symptoms among refugees neither was the number of years since resettlement. The major threats to the mental health of the refugees were the QoL and occupational status. These factors however had even less impact than they did on the non-refugee population. The singular factor to which mental health disparity could be ascribed was therefore in being a refugee. This could be a combination of pre- and post-migration trauma which leaves a lasting mark that persists even after decades in exile. While little can be done to alter refugees' pre-migration experiences, public policies can affect many post-migration experiences in order to mitigate the negative health consequences associated with resettlement. Results of this study point to the need for continued attention to not only the healthcare needs but the welfare, housing, employment and overall quality of life to support the long-term mental health of refugees and non-refugee populations alike.

### Limitations

The cross-sectional nature of the survey did not allow for inferences to be drawn as to causal relationship among variables. Also, the self-reported nature of the data means they were subjective; although participant’s own view is necessary, the question arises whether it is sufficient [46,47]. The refugee population is also a very peculiar one with all its support taken away, thus the possibility of a selection bias in this study. It is however important to note that the majority of refugees in this camp did not take up the option of repatriation to their country of initial origin. The refugee study population was therefore similar in age, gender and country of origin to the original camp profile. Furthermore, the non-refugee population might not be very representative of the wider Nigerian society as more than half of the samples are very highly educated. Despite these limitations, the study provides unique insights into the mental health and quality of life of refugees and Nigerians in Oru-Ijebu which is useful in planning community health services.

## Conclusions

The major factors associated with poor mental health among the refugees were the QoL and occupational status. These factors however had even less impact than they did on the native population. Thus, being a refugee seems to be the most important factor to which mental health disparity could be associated. Results of this study point to the need for continued attention to not only the healthcare needs but the welfare, housing, employment and overall quality of life to support the long-term mental health of refugees and non-refugee populations alike.

## Competing interests

The authors declare that they have no competing interests.

## Authors’ contributions

OOA was involved in the conceptualisation of the study, designed the questionnaire, was involved in statistical analysis and prepared some part of the text. He approved the final manuscript. ETO was involved in the conceptualisation of the study, wrote some part of the text. She approved the final manuscript. OKI was involved in the statistical analysis, wrote part of the text. She approved the final manuscript. OAP was involved in the statistical analysis and wrote part of the text. He approved the final manuscript.
